# A modified QuEChERS sample processing method for the determination of per- and polyfluoroalkyl substances (PFAS) in environmental biological matrices

**DOI:** 10.1016/j.mex.2023.102290

**Published:** 2023-07-20

**Authors:** Xiaoyan Yun, Marie J Kurz, Rominder Suri, Erica R McKenzie

**Affiliations:** a Civil and Environmental Engineering Department, Temple University, Philadelphia, PA 19122, USA; b Academy of Natural Sciences of Drexel University, Philadelphia, PA 19103, USA; c Environmental Sciences Division, Oak Ridge National Laboratory, Oak Ridge, TN 37831, USA

**Keywords:** Biological tissue samples, Contaminant extraction, Sorbents cleanup, PFAS analysis, Matrix interferences, QuEChERS method for PFAS from tissues

## Abstract

QuEChERS (quick, easy, cheap, effective, rugged, and safe) sample processing methods have previously been applied to a range of compounds and matrices. This study presents a modified QuEChERS sample processing method that was validated and employed for 24 per- and polyfluoroalkyl substances (PFAS) for various biological matrices. PFAS are a group of synthetic chemicals that have attracted substantial attention as some compounds are acknowledged to be persistent, toxic, and bioaccumulative. It is crucial to determine PFAS in diverse environmental matrices. Currently, limited sample processing methods for PFAS in biological matrices are available and the majority only focus on a few compounds such as perfluorooctanesulfonic acid (PFOS) and perfluorooctanoic acid (PFOA). Thus, there is a demand to develop a sample processing method which is effective for many commonly tested PFAS compounds in environmental biological samples. In this study, the detailed sample processing procedures and method performance are described. The highlights of this method are:

• The extraction solvent and salts were adjusted for PFAS extraction from environmental biological matrices.
• The modified QuEChERS method is effective for extraction and cleanup from a variety of matrices including algae, plants, invertebrates, amphibians, and fish.

## Graphical abstract

**Figure:**
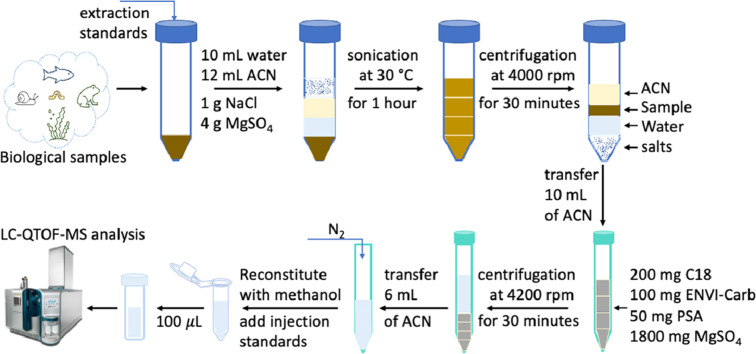

Specifications table

**Table:** 

Subject area	Environmental Science
More specific subject area	PFAS quantification in tissue samples
Name of your method	QuEChERS method for PFAS from tissues
Name and reference of original method	Lacina, O., et al., Simple, high throughput ultra-high performance liquid chromatography/tandem mass spectrometry trace analysis of perfluorinated alkylated substances in food of animal origin: milk and fish. Journal of Chromatography A, 2011. 1218(28): 4312-4321. 10.1016/j.chroma.2011.04.061.
Resource availability	Extraction salts: Sodium chloride (ACS reagent, ≥ 99.0%, Sigma-Aldrich, S9888); Magnesium sulfate (anhydrous, ReagentPlus, ≥ 99.5%, Sigma-Aldrich, M7506).
	Cleanup sorbents: Supelclean ENVI-Carb (Sigma-Aldrich, 57210-U); C18 (endcapped, Agilent Technologies, 5982-5752); Primary secondary amine (PSA, Agilent Technologies, 5982-8382).

## Method details

### Background

The aim of this sample processing method was to effectively extract PFAS from various environmental biological samples, and sufficiently remove interreferences. General information on PFAS and a summary of previously used PFAS extraction methods is provided in Additional Information. In our original method development efforts, we tried a range of sample extraction and cleanup methods. Based on visual assessment of final samples, the previous tested methods did not effectively clean up the samples, and thus were not analyzed (details on tested methods and picture of final samples can be found in S1 and Fig. S1). In this work, a method based on QuEChERS was optimized, validated and applied for quantifying 24 PFAS compounds in a broad range of environmental biological samples. The assessed compounds included 11 perfluoroalkyl carboxylic acids (PFCAs), 7 perfluoroalkane sulfonic acids (PFSAs), 3 fluorotelomer sulfonic acids (FTSs), perfluorooctanesulfonamide (PFOSA) and 2 perfluorooctane sulfonamido acetates (FOSAAs). The key requirement for this sample processing method was to achieve extraction standards recovery of 50%-150% for most compounds, consistent with Department of Defense (DoD) expectations [1].

#### Materials and reagents

• Materials:

 50 mL polypropylene centrifuge tubes, Fisher Scientific, 14-955-239.
 15 mL polypropylene centrifuge tubes, Nest Scientific, 601051.
 2 mL locking polypropylene microcentrifuge tubes, Globe Scientific, 111572LK.
 10-425 WM 500 µL polypropylene vials, Thermo Scientific, 501 354.
 Screw thread 10-425 molded polypropylene septa, Thermo Scientific, 501 357.

• Chemicals:

 PFAS standards, Wellington Laboratories: PFAC-24PAR (analytical standard, native PFAS), MPFAC-24 ES (mass-labeled extraction standard) and MPFAC-C-IS (mass-labeled injection standard).
 Standard reference material (SRM), NIST, SRM 1947-Lake Michigan fish tissue.
 Ammonium acetate, LC/MS Grade, ≥ 99.0%, Sigma-Aldrich, 73594.
 Acetic acid, glacial, Reagent Plus, ≥99%, Sigma-Aldrich, A6283.

• Solvents:

 Acetonitrile, Optima LC/MS Grade, Fisher Chemical, A955-4.
 Water, Optima LC/MS Grade, Fisher Chemical, W64.
 Methanol, Omnisolv LC-MS, MilliporeSigma, MX0486-1.

• Extraction salts:

 Sodium chloride, ACS reagent, ≥ 99.0%, Sigma-Aldrich, S9888.
 Magnesium sulfate, anhydrous, ReagentPlus, ≥ 99.5%, Sigma-Aldrich, M7506.

• Cleanup sorbents:

 Supelclean ENVI-Carb, Sigma-Aldrich, 57210-U.
 C18, endcapped, Agilent Technologies, 5982-5752.
 Primary secondary amine (PSA), Agilent Technologies, 5982-8382.

• Solution preparation:

 Analysis solvent: 0.1% acetic acid in 50%: 50% (volume) methanol: water (i.e., 100 µL acetic acid added to the mixture of 50 mL methanol and 50 mL water).
 24 PFAS spiking solution: methanol diluted PFAC-24 PAR to 50 µg/L.
 Extraction standard (19 ES): methanol diluted MPFAC-24 ES to 25 µg/L.
 Injection standard: 0.1% acetic acid in 50%: 50% methanol: water diluted to 5 µg/L (from methanol diluted MPFAC-C-IS to 50 µg/L).

### Samples description

Two sets of samples and associated results were presented below as follows: 1) method validation, and 2) method application. Collectively, the two samples sets and their respective applications provide a robust assessment of the modified QuEChERS performance.

For 1) method validation, six aliquots of 0.3 g freeze-dried goldfish (*Carassius auratus*) and six aliquots of 0.2 g freeze-dried green lettuce (*Lactuca sativa*) were measured into 50 mL polypropylene (PP) vials. The goldfish and green lettuce were purchased from a local pet store and supermarket, respectively. Spiking solution with 24 PFAS (PFAC-24PAR, 100 µL of 50 µg/L stock; i.e., 5 ng of each compound) was added to each vial and thoroughly mixed. Among the replicates, triplicate goldfish samples and triplicate green lettuce samples were shipped to a Department of Defense (DoD) Environmental Laboratory Accreditation Program (ELAP) accredited laboratory (referred to as “ELAP”) for quantification of 24 PFAS. The ELAP laboratory employed an unknown sample processing method, but is recognized to follow quality control and quality assurance steps that are consistent with DoD expectations [1]. Slight differences existed between the mass labeled extraction standard compounds used by the two laboratories which are detailed in Table S1. The other triplicate goldfish and green lettuce samples were processed and analyzed at Temple University (TU) by a modified QuEChERS sample processing method (referred to as “QuEChERS” or labeled as “TU” in the graphs). Unspiked triplicates of goldfish and green lettuce were also included in TU (QuEChERS) samples; these are not the focus of this study, but results are presented in Table S2.

For 2) method application, samples were collected from streams impacted by historical aqueous film forming foam (AFFF) use, a product that is used to extinguish liquid fuel fires and that can contain elevated PFAS concentrations. Two hundred and eighty-six field collected biological samples (freeze-dried) were included, spanning aquatic species representative of typical Mid-Atlantic stream food webs, from algae to fish. All of the field collected samples were processed by the Academy of Natural Sciences of Drexel University (ANS) using the modified QuEChERS method. TU staff provided a protocol and training for the sample processing; extracted samples were analyzed by TU. All biological samples results are presented on a dry weight-basis, which is the mass of freeze-dried sample that was processed via the modified QuEChERS method; potential residual moisture in the freeze-dried samples was not determined.

### Processing procedures

Each sample (generally containing either 0.2-0.3 g dry weight (freeze-dried), or 2 g wet weight (frozen; i.e., SRM)) was placed in a 50 mL PP centrifuge tube and then spiked with 100 µL of a mass labeled extraction standard (MPFAC-24 ES; stock at 25 µg/L); this equates to 2.5 ng of each extraction standard compound being added to each sample, where a perfect extraction would result in a final concentration in the extract of 1 µg/L. To each sample, 10 mL of LC/MS grade water was added and vortexed for 10 s. Then, 12 mL of LC/MS grade acetonitrile was added and the mixture was vortexed for 30 s to ensure homogenization. Exaction salts (1 g of sodium chloride and 4 g of magnesium sulfate) were added and the vial was vigorously shaken immediately by hand to prevent magnesium coagulation. A batch of samples (maximum of 24 vials) were sonicated at 30°C for one hour in a bath sonicator, followed by centrifugation at 4000 rpm (maximum 3739 Gs) for 30 min to separate different layers (from top to bottom: acetonitrile, sample, water, extraction salts; example photos can be found in Fig. S2).

For cleanup, mixed sorbents of 200 mg of endcapped C18, 100 mg of ENVI-Carb, and 50 mg of primary secondary amine (PSA), along with 1800 mg of magnesium sulfate were loaded into a clean 15 mL PP centrifuge tube. Then 10 mL supernatant from 50 mL extraction PP centrifuge tube (i.e., the top acetonitrile layer) was transferred to the 15 mL PP centrifuge tube. Each 15 mL PP centrifuge tube was manually shaken immediately for 30 s and vortexed for another 30 s; once a batch of samples had been manually created (combined and mixed), the batch of samples was centrifuged at 4200 rpm (maximum 4122 Gs) for 30 min to separate extract and sorbents. A 6 mL aliquot of the cleaned supernatant was transferred to a new 15 mL PP centrifuge tube and the solvent was evaporated under a gentle nitrogen stream to dryness. The dried sample was reconstituted with 0.5 mL of 0.1% acetic acid in methanol and sonicated for 10 min to enhance dissolution. Another 0.5 mL of 0.1% acetic acid in LC/MS grade water was added to the reconstituted sample and vortexed for 10 s. A subsample of 0.8 mL was transferred to a new 2 mL microcentrifuge tube and 0.2 mL of 5 µg/L mass labeled injection standards (MPFAC-C-IS; i.e., 1 ng of each injection standard) was added. The combined sample was vortexed and centrifuged at 13000 rpm (maximum 16200 Gs) for 30 min, then 100 µL was transferred to a PP LC vial for analysis and the remainder was archived. The pH of the sample, as prepared for analysis, was around 4.5, where the slightly acidic pH improves chromatographic separation and sensitivity [2].

**Tips:**

• Recommended quality control samples included in each extraction batch (24 samples) are: two method blanks (empty vials), two laboratory control samples (empty vials spiked with known 24 PFAS), two matrix spike samples (known matrix spiked with known 24 PFAS) and one standard reference material. Taken together this means that a batch contains 7 quality control samples and 17 unknown samples. All quality control samples and unknown samples are processed exactly per the above described processing procedures.
• Ideally, the method blank should be a clean matrix that is processed exactly as a sample. Due to various biological matrices processed in this project, and difficulties in obtaining each clean matrix (i.e., PFAS-free), the empty vials (i.e., initially containing nothing, as purchased), were used as method blanks in each extraction batch. Correspondingly, the laboratory control samples were prepared by spiking known 24 PFAS to empty vials.
• Three centrifugation steps are included in this method. The purpose of the first (50 mL vial) and second (15 mL vial) centrifugation steps are for separating layers. Therefore, the speed and time are flexible, and some adjustment can be made (e.g., based on available centrifuge options). The third (2 mL microcentrifuge tube) centrifugation is performed shortly before analysis, and is intended to remove any residual solids (i.e., further cleanup); though filtration could also remove solids, filter material could act as a source or sink for target PFAS and therefore is not the preferred method. Thus, a relatively high centrifuge speed is recommended.
• Be cautious of the pipette tip depth when transferring supernatant, and avoid the lower layers. If the initial pipetting effort disturbs the layering, then the sample can be re-centrifuged to reestablish the layers and the supernatant pipette transfer can be resumed.
• The procedure can be executed over multiple days, though ideally the sample extraction and cleanup is completed in a single day. The best time to pause sample processing is after transferring 6 mL of the cleaned supernatant to a new 15 mL PP centrifuge tube (i.e., just prior to solvent evaporation via the nitrogen blowdown).
• Biological samples are stored at −20 °C, and extracts are stored at 4 °C. Samples should be kept in 2 mL microcentrifuge tubes and transferred to LC vials shortly before analysis.
• If sample processing and analysis are conducted by two separate laboratories, then the inter-lab sample transfer should occur after reconstituting the samples with 0.5 mL of 0.1% acetic acid and 0.5 mL of 0.1% acetic acid in water. The analysis laboratory will subsample and add mass labeled injection standards. Ideally, the same extraction standard is added to samples prior to extraction and in the calibration curve (i.e., lot and if possible, subsample from the ampule); advanced planning is needed to execute this strategy.

### PFAS analysis

Analysis was performed via liquid chromatography (ExionLC, SCIEX) coupled with quadrupole time-of-flight-mass spectrometry (X500R QTOF, SCIEX) as described by Yun et al. [3]. The LC column (Gemini C-18 column, 50 mm × 3 mm, 3 µm), delay column (Luna C18(2) phase, 30 mm × 3 mm, 5 µm) and guard column (Gemini C18 phase, 4 mm × 2 mm) employed during analysis were all from Phenomenex. Sample injection volume was 10 µL. The MS was operated in the negative electrospray ionization (ESI−) mode using scheduled high-resolution multiple reaction monitoring (scheduled MRMHR) for each compound. PFAS quantitation was accomplished by isotope dilution using mass-labeled extraction standards. Detailed information about analysis conditions and the quality assurance and quality control (QA/QC) are given in S2 and S3, respectively (Table 1).

**Table 1:** Summarized QA/QC and corresponding acceptance criteria.

QA/QC Check	Comments	Acceptance Criteria
Extraction Standards (ES) Recovery	Add to all samples prior to extraction.	Within 50%-150% of the value analyzed in calibration curve.
Injection Standards (IS) Recovery	Added to all samples prior to analysis.	Within 50%-150% of the value analyzed in calibration curve.
Method Validation	Replicate samples are processed and analyzed by two laboratories: TU and ELAP.	Within 70%-130% of the true value or ELAP.
Solvent Blank	0.1% acetic acid in 50%: 50% methanol: water (v/v). Run a solvent blank every six samples.	No analytes detected > ½ LOQ.
Method Blank (MB)	Two MB (empty vials) per extraction batch (24 samples). Processed using the same procedure as samples.	No analytes detected > ½ LOQ.
Laboratory Control Sample (LCS)	Two LCS per extraction batch. Empty vials spiked with all analytes at a mid-level of calibration concentration.	Within 70%-130% of the true value.
Matrix Spike (MS)	Two MS per extraction batch. Samples spiked with all analytes at a mid-level calibration concentration.	Within 70%-130% of the true value.
Standard Reference Material (SRM)	One SRM per extraction batch.	Within 70%-130% of the true value.
Initial Calibration Curve	Linear, minimum of 5 standards concentrations. Include one concentration that is either below all samples or sensitivity limited (e.g., no peak or outside linear range).	The initial calibration curve spanning at least a 20-fold concentration range. R^2^ ≥ 0.98 for each analyte.
Limit of Quantification (LOQ)	Determine the lowest concentration that can be quantified.	The lowest calibration point that is within 70%-130% of the true value and is greater than double of the blanks.
Initial and Continuing Calibration Verification (ICV and CCV)	Analyzed at the beginning of each analysis batch (i.e., ICV), after every 20 samples (i.e., CCV), and at the end of the analysis batch (i.e., CCV).	Must be within 70%-130% of the true value. CCV concentration at the mid-point of the calibration curve.

### Method performance

1) **Method validation**

The modified QuEChERS sample processing method was developed with samples of goldfish (representative fauna matrix) and green lettuce (representative flora matrix) to evaluate extraction solvent, sorbent selection and amount, and optimize processing procedures. Previous results suggested that 1) formic acid affected PFAS partitioning among the extraction phases; after adding water, when acetonitrile amended with 2% formic acid was used as the extraction solvent it caused low recovery for all compounds in all samples. 2) PSA is not suitable to be used in large quantities for PFAS samples, although it is commonly used in traditional QuEChERS cleanup; applying 400 mg PSA as one of the sorbents resulted in low recovery in all samples without matrix (two method blanks (empty vials) and two laboratory control samples (empty vials spiked with known 24 PFAS)) and in some samples with matrix. 3) One hour sonication facilitated extraction and ten minute sonication after reconstitution are necessary to increase sample dispersal and obtain homogenous samples so as to increase extraction recovery and precision. The duplicate method blanks (empty vials) met the quality control measures of non-detectable or low contamination (< ½ limit of quantitation (LOQ)), and the average spike recoveries of duplicate laboratory control samples (empty vials spiked with known 24 PFAS) were 76% - 117%. The LOQs and spike recoveries of each compound can be found in Table S5. In the current study, the samples were extracted from a freeze-dried form by two independent laboratories, and LOQs were presented on a dry weight-basis in this manuscript; the LOQ values were comparable or slightly higher than the reporting limit from ELAP assessment. The wet weight based LOQs by this method can be found in the related research articles [3,4].

**Fig. 1:**
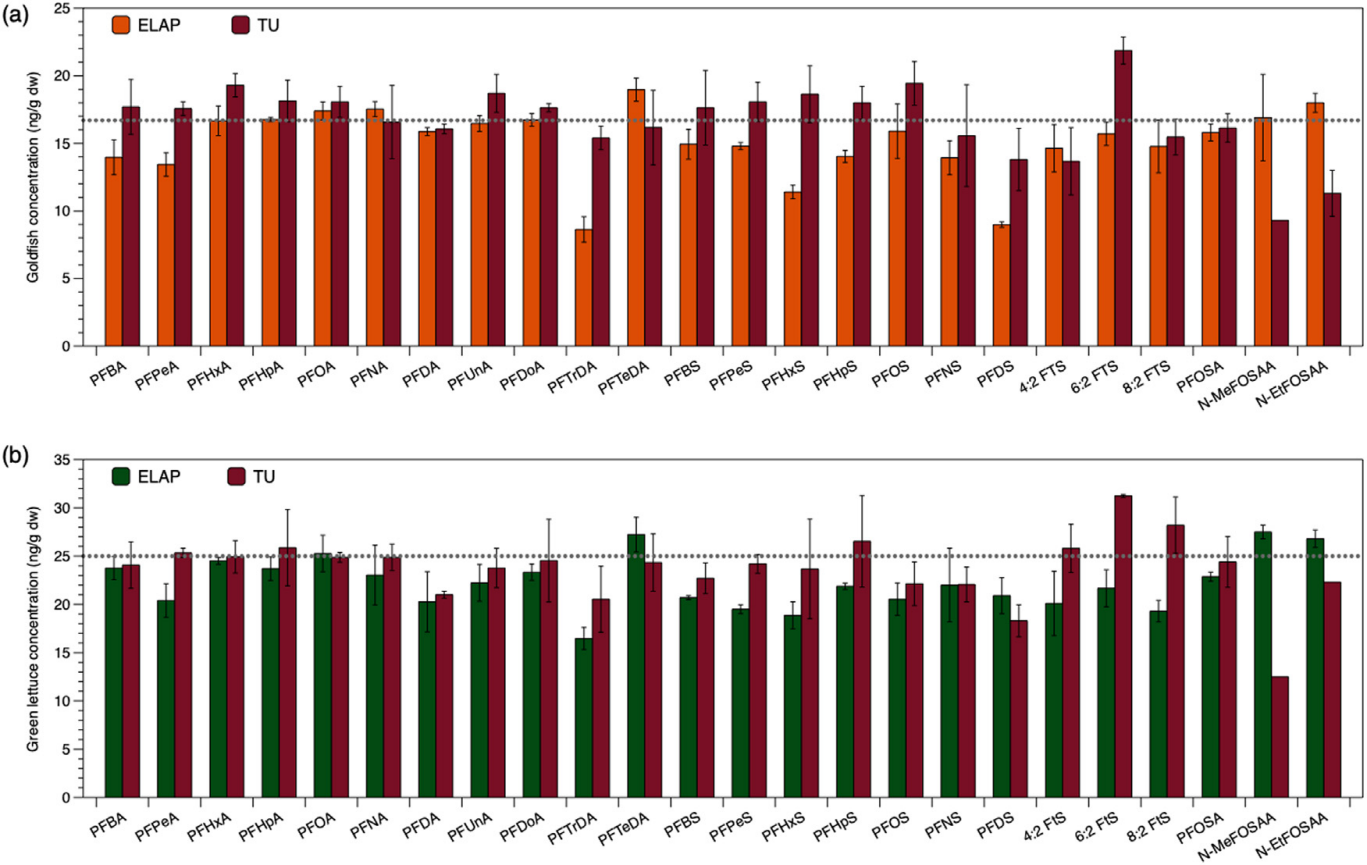
The average concentrations of 24 native PFAS, as determined based on the isotope dilution method, analyzed by ELAP and TU (QuEChERS) for (a) triplicate goldfish and (b) triplicate green lettuce, where the grey dotted line represents the nominal spiked concentrations. Bars show mean ± standard deviation.

The method was validated through comparing triplicates of each matrix, with evaluation based on spiked concentration and extraction standard recovery. The average concentrations of goldfish and green lettuce determined by ELAP (proprietary extraction method) and TU (QuEChERS) are shown in Fig. 1, and related data are listed in Table S6. Due to the weakness of this method for N-MeFOSAA and N-EtFOSAA (discussed in next paragraph), the validation focused on 22 analytes. Generally, most of the analyte concentrations were within 30% of the nominal concentrations for both laboratories, which was the acceptance criteria. However, TU (QuEChERS) analyzed concentrations were more similar to nominal concentrations than ELAP reported concentrations: 103 ± 11% (goldfish) and 97 ± 11% (green lettuce) by TU, and 89 ± 15% (goldfish) and 87 ± 10% (green lettuce) by ELAP. For the TU (QuEChERS) method, 21 of the 22 analytes in goldfish (except 6:2 FTS, 131% of nominal concentration), and all analytes in green lettuce were within 30% of the nominal concentration. For the ELAP method, results indicated 19 and 21 of the 22 analytes in goldfish and green lettuce, respectively were within 30% of the nominal concentrations. In some cases, the ELAP results were bias low. The goldfish samples were laboratory spiked with known nominal concentration of 16.7 ng/g dw for each compound, where analytical standards were used as the spiking solution (i.e., accurate concentration of each analyte is expected). Concentrations reported by ELAP are notably below the nominal concentration for PFTrDA, PFHxS and PFDS in goldfish, and PFTrDA in green lettuce; this is especially true for PFTrDA and PFDS, which do not have corresponding mass-labelled extraction standards for quantification (either method; Table S1). When compared with nominal PFTrDA, PFHxS and PFDS concentrations in goldfish samples, the TU (QuEChERS) analysis results achieved 92%, 111% and 83%, respectively; whereas 51%, 68% and 54% for the ELAP analysis.

**Fig. 2:**
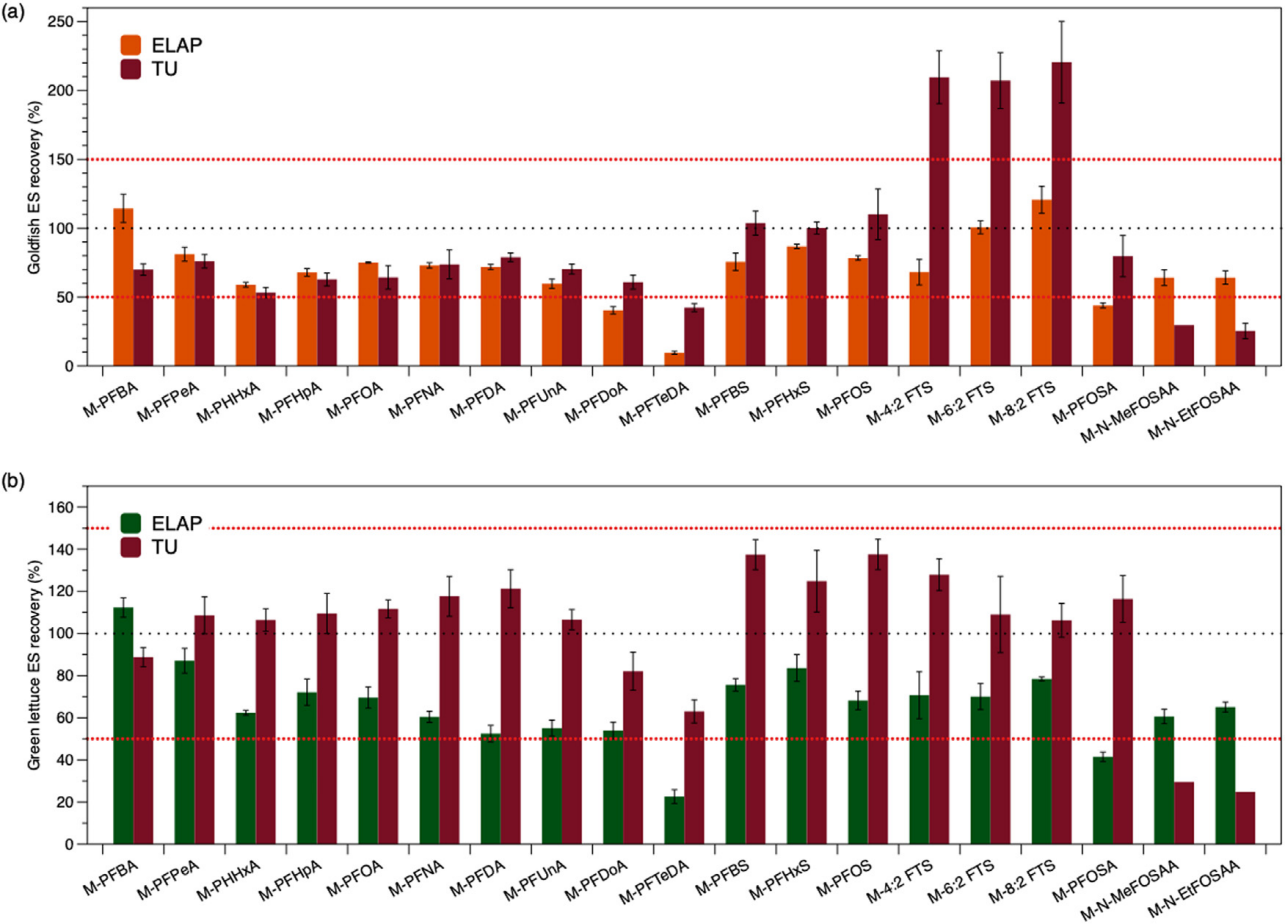
The average mass-labeled extraction standards (ES) recovery analyzed by ELAP and TU (QuEChERS) for (a) triplicate goldfish and (b) triplicate green lettuce, where the red dotted lines represent the acceptance criteria (50%- 150%), the black dotted line (100%) represents the optimal reference. The extraction standard includes 19 mass-labeled analytes, compared to 24 native PFAS quantified by the method (see Table S1). Bars show mean ± standard deviation.

The average extraction standard recovery results reflecting the performance from TU (QuEChERS) and ELAP (proprietary extraction method) for the 19 mass labeled compounds are compared in Fig. 2, and related data are listed in Table S7. More severe matrix effects was reflected in goldfish compared with green lettuce for both laboratories. Overall, lower extraction standard recoveries were obtained in goldfish samples (i.e., 74 ± 20% (TU) and 69 ± 25% (ELAP) for extraction standard recovery of 13 PFAA compounds). While for green lettuce samples, the average extraction standard recoveries of TU (QuEChERS) were notably better than ELAP (i.e., 109 ± 21% (TU) and 67 ± 21% (ELAP) for extraction standard recovery of 13 PFAA compounds). Results indicated that the extraction standard recoveries were matric specific. For example, the FTS extraction standard recoveries were above the acceptable range (50%-150%) for the goldfish samples and were notably higher than the green lettuce samples (i.e., approximately two-fold difference), though both matrices were processed and analyzed identically and concurrently at TU (QuEChERS).

Limitations were observed for N-MeFOSAA and N-EtFOSAA for samples processed using the modified QuEChERS method which resulted in low extraction standard recoveries (i.e., d3-N-MeFOSAA and d5-N-EtFOSAA: less than 30% in all samples). The low extraction standard recovery suggests that one or more of the following was occurring: 1) extraction from the matrix was incomplete, 2) the compounds were extracted but then lost to sample processing components (e.g., sorbents, vaporization during the nitrogen blowdown), and 3) the compounds were transformed during the samples processing steps. Data from the validation and QA/QC samples suggest that either 1) or 2) rather than 3) likely contribute to the low extraction standard recovery of d3-N-MeFOSAA and d5-N-EtFOSAA. The sample processing method does not create an environment likely to cause chemical transformations, and none of the targeted compounds were detected as transformation products. Based on the isotope dilution approach, the calculated native compound concentrations of N-MeFOSAA and N-EtFOSAA are closer to the expected value (Fig. 1) though did not meet the recovery criteria. In other extraction rounds, low recovery of d3-N-MeFOSAA and d5-N-EtFOSAA were consistently observed, but in some cases the laboratory control samples and matrix spike samples met the criteria for the native compounds, supporting that the mass-labeled and native compounds experience similar loss mechanisms. While isotope dilution approach can account for some changes (e.g., discussed for FTS in the next paragraph), the uncertainty increases as the recovery deviates farther from ideal, likely resulting in the low N-MeFOSAA and N-EtFOSAA concentrations reported in Fig. 1. The mass spectrometer used in this study had lower sensitivity for N-MeFOSAA and N-EtFOSAA (native and mass-labeled) which makes the isotope dilution method less robust; higher extraction standard concentrations can be used to address this analytical limitation, but won't address the low recoveries arising from the sample processing method.

In the TU (QuEChERS) analysis, the high extraction standards recovery of three mass-labeled FTSs in goldfish samples (> 150%, Fig. 2) were attributed to the goldfish matrix ionization enhancement effect, though isotope dilution determined concentrations were comparable to nominal concentrations (82%, 131% and 93% of nominal concentrations of 4:2, 6:2 and 8:2 FTS, respectively). The extraction standard recovery of three mass-labeled FTSs from method blanks and laboratory control samples were within acceptance criteria. From some matrix spike samples and the unknown samples, the FTS ES recoveries were higher than 150% (e.g., goldfish). This suggests the high extraction standard recovery was caused by the specific matrix. Based on observation, compound ionization was affected by matrix components for the FTS compounds and impacted both mass-labelled and native analytes similarly. Therefore, while the FTS compound results are not considered quantitative in goldfish samples, reasonable estimates of the concentrations were achieved (i.e., similar to nominal concentrations and ELAP analyzed concentrations). Generally, the modified QuEChERS method obtained reasonable extraction standards recovery and analytes concentrations relative to the external laboratory concentrations and known spiked concentrations.

Due to the complexity of the biological samples, some targeted compounds can be affected by the specific matrix. Based on our observations and comparisons, extraction of freeze-dried samples reduces matric effect (based on extraction standard recovery values) compared to extraction of wet samples. The improved extraction performance is more notable for some long chain length PFAS. In addition, inclusion of matrix spike samples is useful to evaluate the matrix effects as this provides information on mass-labeled and native analytes; if mass-labeled and native analytes are similarly affected (e.g., FTSs in goldfish), the isotope dilution can provide a reliable estimate. Furthermore, matrix effects can be reduced by increasing the extraction standard concentrations and decreasing injection volume, though this can create sensitivity issues (i.e., < LOQ) for compounds that are present in lower concentrations and /or for which the sensitivity is analytically lower (i.e., ionization and fragmentation).

2) **Method application**

Besides goldfish (*Carassius auratus*) and green lettuce (*Lactuca sativa*), the modified QuEChERS method was also found to performed well for standard reference material (SRM 1947-Lake Michigan Fish Tissue), and laboratory cultured macroinvertebrates including aquatic worms (*Lumbriculus variegatus*), freshwater mussels (*Elliptio complanata*) and snails (*Physella acuta*); while the method validation performed for the current manuscript included 24 compounds, other applications may consider only a subset of analytes (e.g., publication from our group [3,4]). Consequently, the method was adopted by a second laboratory to process field collected biological samples comprising a wide range of freshwater stream and riparian species. These samples were subsequently analyzed at TU.

PFAS samples had not previously been processed at the second laboratory, the Academy of Natural Sciences (ANS), nor by any of the ANS personnel. Several steps were taken to ensure effective and comparable application of the modified QuEChERS method. A dedicated, enclosed laboratory space was set aside for PFAS work and cleaned thoroughly with soap and then reagent-grade methanol prior to use. Equipment, laboratory, and deionized water blanks from ANS were analyzed by TU to confirm background PFAS concentrations were acceptably low prior to initiating any PFAS sample processing. TU shared both the method protocol and the supplier details of all materials used in the method. Wherever possible, ANS purchased the identical equipment, consumables, reagents and standards as TU; major exceptions being existing centrifuge, vacuum, and nitrogen blowdown units that were repurposed after thorough cleaning. Execution of the method was done in close communication with TU, including initial sharing of the extraction procedure, a visit by ANS personnel to TU to observe the method in process, and video calls (due to COVID restrictions) to confirm the setup and progress at ANS. Extracted samples were transported by ANS personnel to TU for subsequent analysis. In addition to the samples and recommended quality control samples, PFAS standards used at ANS were also analyzed at TU.

Due to the wide variety of samples, the extraction standards recoveries listed in Table 2 have been categorized as algae, vascular plants, macroinvertebrates, amphibians, and fish. It is noteworthy each category is composed of different species/tissues. For example, aquatic plants include various submerged, floating and emergent species, with some species separated into leaves and stems plus roots. In the category of non-insect macroinvertebrates, extracted species include crayfish, snails (shelled and unshelled), clams (shelled), earthworms, mussels (shelled) and aquatic worms. In the largest category of fish (n=148), 17 species were extracted including white sucker (*Catostomus commersonii*, n=13), green sunfish (*Lepomis cyanellus*, n=13), American eel (*Anguilla rostrata*, n=13), tessellated darter (*Etheostoma olmstedi*, n=12), redbreast sunfish (*Lepomis auritus*, n=11), common shiner (*Luxilus cornutus*, n=11), creek chub (*Semotilus atromaculatus*, n=10), eastern blacknose dace (*Rhinichthys atratulus*, n=10), yellow bullhead catfish (*Ameiurus natalis*, n=9), banded killifish (*Fundulus diaphanus*, n=9), spotfin shiner (*Cyprinella spiloptera*, n=8), pumpkinseed (*Lepomis gibbosus*, n=7), bluegill (*Lepomis macrochirus*, n=6), largemouth bass (*Micropterus salmoides*, n=6), spottail shiner (*Notropis hudsonius*, n=4), western mosquitofish (*Gambusia affinis*, n=3), and rock bass (*Ambloplites rupestris*, n=3). Except where noted above, all field samples were processed as whole body. All samples were freeze-dried and then homogenized, with some samples comprised of individual organisms and some of composites, based on organism size.

**Table 2:** Extraction standards recovery (%) in a broad range of field matrices processed by modified QuEChERS^a^.

Overall, the modified QuEChERS achieved acceptable recoveries for commonly detected perfluoroalkyl acids (PFAAs), matrix enhancement on FTS compounds, inhibition on PFOSA, and poor recoveries for N-MeFOSAA and N-EtFOSAA (Table 2). Given these were field collected samples (i.e., not spiked), a comparison to an expected concentration is not possible (in contrast with method validation effort), and extraction standard recovery is the best endpoint for comparison. Although the application involved a large number of various matrices, results were comparable to method validation samples, with matrix effects being species and compounds specific.

## CRediT authorship contribution statement

**Xiaoyan Yun:** Conceptualization, Methodology, Investigation, Formal analysis, Visualization, Writing – original draft. **Marie J Kurz:** Funding acquisition, Writing – original draft, Resources, Project administration, Supervision. **Rominder Suri:** Funding acquisition, Writing – review & editing. **Erica R McKenzie:** Funding acquisition, Conceptualization, Writing – review & editing, Resources, Supervision.

## Declaration of Competing Interest

The authors declare that they have no known competing financial interests or personal relationships that could have appeared to influence the work reported in this paper.

## Data Availability

Data will be made available on request.

## Appendix Supplementary materials

Image, application 1

Image, application 2
