# Supplementary material for: A modified QuEChERS sample processing method for the determination of per- and polyfluoroalkyl substances (PFAS) in environmental biological matrices
Source: MethodsX. 2023 Jul 20;11:102290. doi: 10.1016/j.mex.2023.102290 (PMC10413343; doi:10.1016/j.mex.2023.102290)
Supplement: Supplementary file 1 [file mmc1.docx]

**Supplementary Material**

Xiaoyan Yun^a^, Marie J Kurz^b,c^, Rominder Suri^a^, Erica R McKenzie^a, *^

^a^ Civil and Environmental Engineering Department, Temple University, Philadelphia, PA, 19122, USA

^b^ Academy of Natural Sciences of Drexel University, Philadelphia, PA, 19103, USA

^c^ Environmental Sciences Division, Oak Ridge National Laboratory, Oak Ridge, TN, 37831, USA. This manuscript has been authored by UT-Battelle, LLC, under contract DE-AC05-00OR22725 with the US Department of Energy (DOE). The publisher acknowledges the US government license to provide public access to these results in accordance with the DOE Public Access Plan (<https://energy.gov/downloads/doe-public-access-plan>).

^*^**Corresponding author:** Erica R McKenzie, Civil and Environmental Engineering Department, Temple University, Philadelphia, PA, 19122, USA

E-mail address: [ermckenzie@temple.edu](mailto:ermckenzie@temple.edu)

**Table of Contents**

S1 Previous tested methods…………………………………………………………………………….3

Figure S1 Visualizations of different methods for goldfish samples ………………………………….3

Table S1 Analytes and associated extraction standards that were being utilized in method validation……………………………………………………………………….……………………....….4

Table S2 PFAS concentrations and associated extraction standards recovery in unspiked samples…………………. ……………………………………………………………………….……….5

Figure S2 Example photos of actual samples before adding extraction salts and after the first centrifugation (show different layers) …………………………………………. ……………………….6

S2 PFAS analysis………………………………………………………………………………….…...…7

Table S3 LC method solvent gradient profile………………………………………………….……….7

Table S4 MS method for PFAS identification and quantification………………………….……….…7

S3 Quality assurance and quality control………………………………………………………….……9

Table S5 The LOQs and spike recoveries of laboratory control samples…………………………10

Table S6 PFAS concentrations analyzed for the method validation…………………………….…11

Table S7 Extraction standards recovery analyzed for the method validation….……………….….12

**S1. Previous tested methods**

At the very beginning we tried four commonly used methods in literature to our representative matrix goldfish and green lettuce: M1) alkaline digestion extraction (potassium hydroxide in methanol) followed by SPE cartridge cleanup, M2) acetonitrile extraction followed by SPE cartridge cleanup, M3) alkaline digestion (potassium hydroxide in methanol) followed by ENVI-Carb cleanup and SPE cartridge cleanup, M4) acetonitrile extraction followed by ENVI-Carb cleanup SPE cartridge cleanup. And found SPE cartridge can’t remove pigments, the extracts were green for lettuce samples. For goldfish samples, after SPE cartridge cleanup or ENVI-Carb and SPE cartridge cleanup, samples still looked dirty/murky (Figure S1). Thus, we didn’t run these samples since visually poor cleanup by above methods.


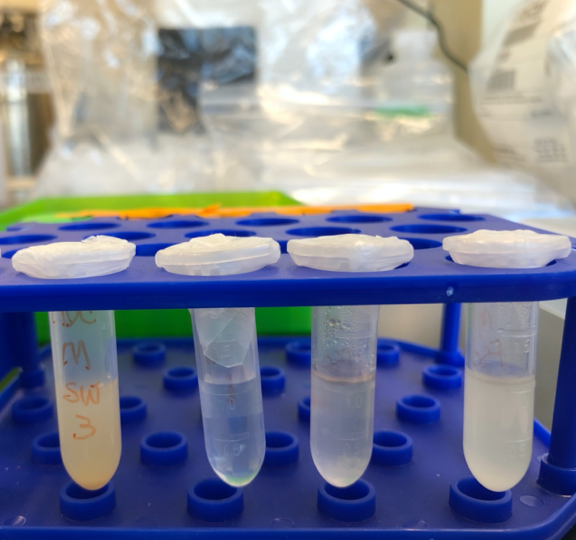


From left to right:

- M1) alkaline digestion extraction (potassium hydroxide in methanol) followed by SPE cartridge cleanup
- method blank (as reference)
- M3) alkaline digestion extraction (potassium hydroxide in methanol) followed by ENVI-Carb and SPE cartridge cleanup
- M4) acetonitrile extraction followed by ENVI-Carb and SPE cartridge cleanup

(* M2) acetonitrile extraction followed by SPE cartridge cleanup is missing because all SPE cartridges were clogged by acetonitrile extracts)

**Figure S1** Visualizations of different methods for goldfish samples.

**Table S1** Analytes and associated available mass labeled extraction standards that were being utilized in method validation.

| **Analyte** | **CAS Number** | **ELAP** ^a^ | **TU** ^b^ |
| --- | --- | --- | --- |
| PFBA | 375-22-4 | M3PFBA | M4PFBA |
| PFPeA | 2706-90-3 | M3PFPeA | M5PFPeA |
| PFHxA | 307-24-4 | M2PFHxA | M5PFHxA |
| PFHpA | 375-85-9 | M4PFHpA | M4PFHpA |
| PFOA | 335-67-1 | M2PFOA | M8PFOA |
| PFNA | 375-95-1 | M5PFNA | M9PFNA |
| PFDA | 335-76-2 | M2PFDA | M6PFDA |
| PFUnA | 2058-94-8 | M2PFUnA | M7PFUnA |
| PFDoA | 307-55-1 | M2PFDoA | M2PFDoA |
| PFTrDA | 72629-94-8 | ------ | ------^c^ |
| PFTeDA | 376-06-7 | M2PFTeDA | M2PFTeDA |
| PFBS | 375-73-5 | M3PFBS | M3PFBS |
| PFPeS | 2706-91-4 | ------ | ------^c^ |
| PFHxS | 355-46-4 | M3PFHxS | M3PFHxS |
| PFHpS | 375-92-8 | ------ | ------^c^ |
| PFOS | 1763-23-1 | M8PFOS | M8PFOS |
| PFNS | 68259-12-1 | ------ | ------^c^ |
| PFDS | 335-77-3 | ------ | ------^c^ |
| 4:2 FTS | 757124-72-4 | M2-4:2 FTS | M2-4:2 FTS |
| 6:2 FTS | 27619-97-2 | M2-6:2 FTS | M2-6:2 FTS |
| 8:2 FTS | 39108-34-4 | M2-8:2 FTS | M2-8:2 FTS |
| PFOSA | 754-91-6 | M8PFOSA | M8PFOSA |
| N-MeFOSAA | 2355-31-9 | d3-N-MeFOSAA | d3-N-MeFOSAA |
| N-EtFOSAA | 2991-50-6 | d5-N-EtFOSAA | d5-N-EtFOSAA |

^a^ Extraction standards used by the DoD Environmental Laboratory Accreditation Program (ELAP) accredited commercial laboratory.

^b^ Extraction standards used by Temple University (TU) processed via the modified QuEChERS method.

^c^ Alternate mass-labeled extraction standards were used for quantitation; for PFTrDA, M2PFDoA was used; for PFPeS, M3PFBS was used; for PFHpS, M3PFHxS was used; for both PFNS and PFDS, M8PFOS was used.

**Table S2** PFAS concentrations and associated extraction standards recovery (%) in unspiked samples analyzed by TU.

|  |  | **Concentrations (ng/g dw)** | |  | **Extraction standards recovery (%)** | |
| --- | --- | --- | --- | --- | --- | --- |
| **Analyte** |  | Goldfish | Green lettuce |  | Goldfish | Green lettuce |
| PFBA |  | < 4.2 | < 6.25 |  | 73.9±1.9 | 82.7±8.0 |
| PFPeA |  | < 6.7 | < 10 |  | 70.3±5.0 | 103.9±12.2 |
| PFHxA |  | < 4.2 | < 6.25 |  | 52.1±7.9 | 106.1±20.2 |
| PFHpA |  | < 0.8 | < 1.25 |  | 66.6±1.8 | 107.5±6.7 |
| PFOA |  | < 4.2 | < 6.25 |  | 68.5±4.7 | 107.7±10.3 |
| PFNA |  | < 4.2 | < 6.25 |  | 80.2±5.2 | 110.9±12.3 |
| PFDA |  | < 4.2 | ND |  | 77.2±6.0 | 113.6±8.5 |
| PFUnA |  | < 4.2 | ND |  | 68.9±6.0 | 102.3±16.4 |
| PFDoA |  | < 4.2 | ND |  | 64.1±7.2 | 77.3±12.7 |
| PFTrDA |  | < 4.2 | ND |  | ------ | ------ |
| PFTeDA |  | ND | ND |  | 44.0±6.2 | 58.4±10.4 |
| PFBS |  | ND | ND |  | 109.8±2.5 | 119.7±13.9 |
| PFPeS |  | < 6.7 | ND |  | ------ | ------ |
| PFHxS |  | ND | ND |  | 105.2±14.6 | 122.7±30.0 |
| PFHpS |  | ND | ND |  | ------ | ------ |
| PFOS |  | 5.9±1.6 | ND |  | 101.9±9.7 | 128.7±7.9 |
| PFNS |  | ND | ND |  | ------ | ------ |
| PFDS |  | ND | ND |  | ------ | ------ |
| 4:2 FTS |  | ND | ND |  | 202.4±26.2 | 129.7±2.6 |
| 6:2 FTS |  | < 4.2 | ND |  | 205.1±63.6 | 93.8±21.9 |
| 8:2 FTS |  | ND | ND |  | 174.8±23.9 | 79.2±15.4 |
| PFOSA |  | ND | ND |  | 70.4±2.9 | 94.8±19.3 |
| N-MeFOSAA |  | ND | ND |  | * | * |
| N-EtFOSAA |  | ND | ND |  | * | * |

ND: not detected.

< x: a peak was observed but less than the compound specific limit of quantitation (LOQ).

* Extraction standard was not detected or recovery < 30%.

Grey highlights extraction standard recovery < 50% or > 150%.


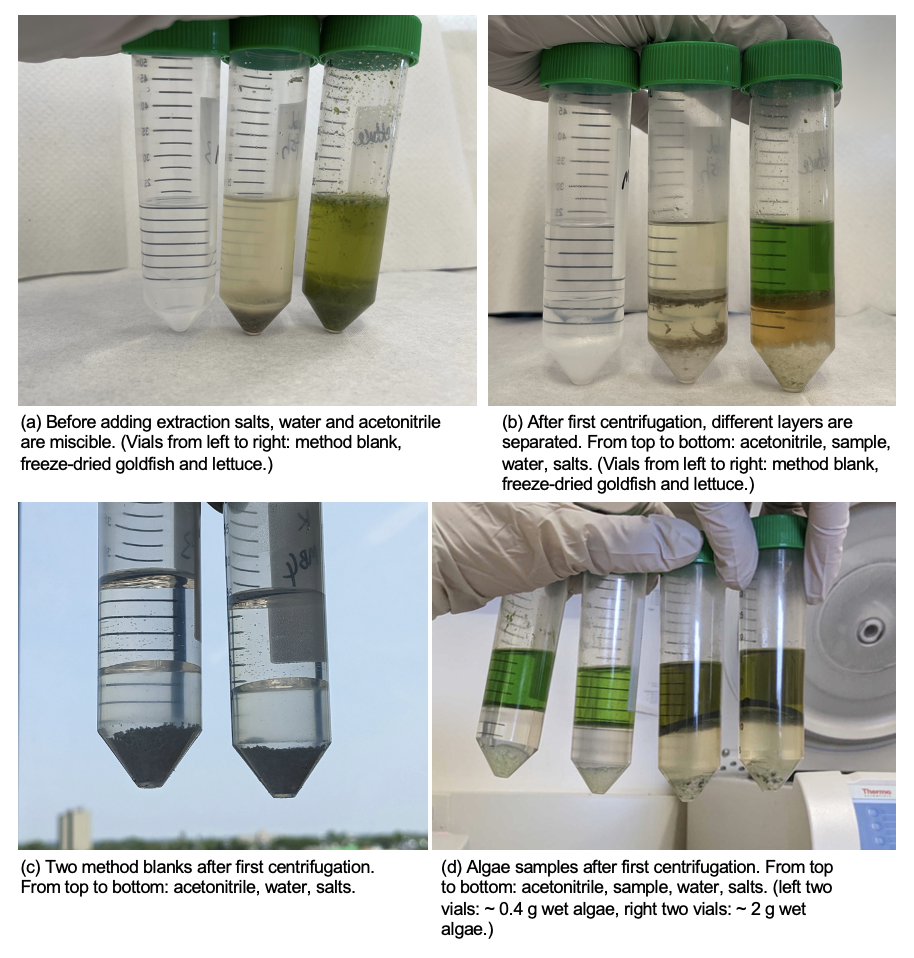


**Figure S2** Example photos of actual samples before adding extraction salts and after the first centrifugation (show different layers).

**S2. PFAS analysis**

The column temperature was maintained at 40°C. The analytes were eluted using 10 mM ammonium acetate in LC/MS grade water (Solvent A) and 10 mM ammonium acetate in LC/MS grade methanol (Solvent B) as mobile phases at a flow rate of 0.5 mL/min. The electrospray ionization capillary voltage was 4500 V with source temperature of 500 °C. The mass spectrometer was operated in negative electrospray ionization (ESI−) mode using scheduled high-resolution multiple reaction monitoring (scheduled MRMHR) for each compound; generally, two transitions were monitored for each targeted analyte. Further details about LC solvent gradient profile and MS mass transition ions, declustering potential and collision energy are listed in Table S3 and Table S4.

**Table S3** LC method solvent gradient profile.

| **Time**  **(min)** | **Flow rate**  **(mL/min)** | **% Solvent A**  **(10mM ammonium acetate in Water)** | **% Solvent B**  **(10 mM ammonium acetate in Methanol)** |
| --- | --- | --- | --- |
| 0.00 | 0.5 | 99 | 1 |
| 0.25 | 0.5 | 99 | 1 |
| 0.50 | 0.5 | 50 | 50 |
| 4.50 | 0.5 | 1 | 99 |
| 8.50 | 0.5 | 1 | 99 |
| 10.0 | 0.5 | 99 | 1 |
| 13.0 | 0.5 | 99 | 1 |

**Table S4** MS method for PFAS identification and quantification.

| **Analytical**  **compound** | | **Precursor ion (Da)** | | **Fragment ion (Da)** | | **Declustering**  **potential (V)** | | **Collision**  **Energy (V)** | | **Retention**  **Time (min)** | |
| --- | --- | --- | --- | --- | --- | --- | --- | --- | --- | --- | --- |
| PFBA | | 213 | | 169 | | -40 | | -10 | | 2.82 | |
| M4PFBA | | 217 | | 172 | | -40 | | -10 | | 2.82 | |
| M3PFBA | | 216 | | 172 | | -25 | | -10 | | 2.82 | |
| PFPeA | | 263 | | 219 | | -35 | | -10 | | 3.25 | |
| M5PFPeA | | 268 | | 223 | | -25 | | -10 | | 3.25 | |
| PFBS | | 299 | | 80 | | -80 | | -45 | | 3.29 | |
| PFBS_2 | | 299 | | 99 | | -80 | | -35 | | 3.29 | |
| M3PFBS | | 302 | | 80 | | -30 | | -75 | | 3.29 | |
| 4:2 FTS | | 327 | | 307 | | -100 | | -25 | | 3.64 | |
| 4:2 FTS_2 | | 327 | | 81 | | -100 | | -35 | | 3.64 | |
| M2-4:2 FTS | | 329 | | 81 | | -25 | | -35 | | 3.64 | |
| PFHxA | | 313 | | 269 | | -25 | | -10 | | 3.68 | |
| PFHxA_2 | | 313 | | 119 | | -25 | | -25 | | 3.68 | |
| M5PFHxA | | 318 | | 273 | | -40 | | -10 | | 3.68 | |
| PFPeS | | 349 | | 80 | | -50 | | -55 | | 3.68 | |
| PFPeS_2 | | 349 | | 99 | | -50 | | -45 | | 3.68 | |
| PFHpA | | 363 | | 319 | | -55 | | -10 | | 4.06 | |
| PFHpA_2 | | 363 | | 169 | | -55 | | -20 | | 4.06 | |
| M4PFHpA | | 367 | | 322 | | -25 | | -10 | | 4.06 | |
| PFHxS | | 399 | | 80 | | -55 | | -60 | | 4.06 | |
| PFHxS_2 | | 399 | | 99 | | -55 | | -45 | | 4.06 | |
| M3PFHxS | | 402 | | 80 | | -30 | | -60 | | 4.06 | |
| 6:2 FTS | | 427 | | 407 | | -55 | | -30 | | 4.39 | |
| 6:2 FTS_2 | | 427 | | 81 | | -55 | | -35 | | 4.39 | |
| M2-6:2FTS | | 429 | | 81 | | -30 | | -45 | | 4.39 | |
| PFOA | | 413 | | 369 | | -25 | | -15 | | 4.39 | |
| PFOA_2 | | 413 | | 169 | | -25 | | -25 | | 4.39 | |
| M8PFOA | | 421 | | 376 | | -70 | | -15 | | 4.39 | |
| M2PFOA | | 415 | | 370 | | -40 | | -10 | | 4.39 | |
| PFHpS | | 449 | | 80 | | -70 | | -60 | | 4.39 | |
| PFHpS_2 | | 449 | | 99 | | -70 | | -45 | | 4.39 | |
| PFNA | | 463 | | 419 | | -30 | | -15 | | 4.70 | |
| PFNA_2 | | 463 | | 169 | | -30 | | -20 | | 4.70 | |
| M9PFNA | | 472 | | 427 | | -40 | | -10 | | 4.70 | |
| PFOS | | 499 | | 80 | | -50 | | -75 | | 4.66 | |
| PFOS_2 | | 499 | | 99 | | -50 | | -50 | | 4.66 | |
| M8PFOS | | 507 | | 80 | | -25 | | -75 | | 4.66 | |
| M4PFOS | | 503 | | 80 | | -30 | | -95 | | 4.66 | |
| PFNS | | 549 | | 80 | | -25 | | -75 | | 4.94 | |
| PFNS_2 | | 549 | | 99 | | -25 | | -60 | | 4.94 | |
| 8:2 FTS | | 527 | | 507 | | -115 | | -35 | | 4.98 | |
| 8:2 FTS_2 | | 527 | | 81 | | -115 | | -55 | | 4.98 | |
| M2-8:2FTS | | 529 | | 81 | | -25 | | -50 | | 4.98 | |
| PFDA | | 513 | | 469 | | -65 | | -20 | | 4.98 | |
| PFDA_2 | | 513 | | 219 | | -65 | | -20 | | 4.98 | |
| M6PFDA | | 519 | | 474 | | -80 | | -10 | | 4.98 | |
| M2PFDA | | 515 | | 470 | | -40 | | -15 | | 4.98 | |
| PFOSA | | 498 | | 78 | | -115 | | -45 | | 4.99 | |
| PFOSA_2 | | 498 | | 64 | | -115 | | -135 | | 4.99 | |
| M8PFOSA | | 506 | | 78 | | -30 | | -45 | | 4.99 | |
| N-MeFOSAA | | 570 | | 419 | | -30 | | -25 | | 5.10 | |
| N-MeFOSAA_2 | | 570 | | 483 | | -30 | | -20 | | 5.10 | |
| d3-N-MeFOSAA | | 573 | | 419 | | -35 | | -25 | | 5.10 | |
| PFDS | | 599 | | 80 | | -35 | | -70 | | 5.19 | |
| PFDS_2 | | 599 | | 99 | | -35 | | -60 | | 5.19 | |
| PFUnA | | 563 | | 519 | | -60 | | -15 | | 5.18 | |
| PFUnA_2 | | 563 | | 269 | | -60 | | -20 | | 5.18 | |
| M7PFUnA | | 570 | | 525 | | -85 | | -15 | | 5.18 | |
| N-EtFOSAA | | 584 | | 419 | | -85 | | -25 | | 5.22 | |
| N-EtFOSAA_2 | | 584 | | 526 | | -85 | | -25 | | 5.22 | |
| d5-N-EtFOSAA | | 589 | | 419 | | -80 | | -25 | | 5.22 | |
| PFDoA | | 613 | | 569 | | -45 | | -15 | | 5.40 | |
| PFDoA_2 | | 613 | | 169 | | -45 | | -40 | | 5.40 | |
| M2PFDoA | | 615 | | 570 | | -30 | | -15 | | 5.40 | |
| PFTrDA | | 663 | | 619 | | -25 | | -20 | | 5.58 | |
| PFTrDA_2 | | 663 | | 169 | | -25 | | -40 | | 5.58 | |
| PFTeDA | | 713 | | 669 | | -30 | | -20 | | 5.74 | |
| PFTeDA_2 | | 713 | | 169 | | -30 | | -35 | | 5.74 | |
| M2PFTeDA | | 715 | | 670 | | -50 | | -20 | | 5.74 | |

**S3. Quality assurance and quality control (QA/QC)**

During sample processing, two method blanks (empty vials), two laboratory control samples (empty vials spiked with certain amount of 24 PFAS), two matrix spike samples (pre-quantified freeze-dried goldfish or filed samples spiked with certain amount of 24 PFAS) and one standard reference material (SRM) were extracted with each batch to assess sample processing contamination and matrix effects.

During PFAS analysis, a laboratory solvent blank (0.1% acetic acid in 50%: 50% methanol: water) was analyzed every 6 samples. Initial calibration verification (ICV) accuracy was verified by analyzing an analytical standard (different product than used for the calibration curve); the ICV was analyzed following the calibration curve and prior to any samples. Continuous calibration verification (CCV) samples were analyzed after every 20 samples, and at the end of the analysis batch.

PFAS were quantified by isotope dilution using a calibration curve comprising 15 points spanning from 10 ng/L to 50 μg/L. The limit of quantitation (LOQ) was batch and compound-specific and defined as the lowest point of the calibration curve calculated to be within 30% of its true value.

**Table S5** The LOQs and spike recoveries of laboratory control samples (no matrix, empty vials spiked with known 24 PFAS).

|  |  | **LOQ (ng/g dw)** | |  | **LCS recovery (%)** ^a^ |
| --- | --- | --- | --- | --- | --- |
| **Analyte** |  | Goldfish | Green lettuce |  |  |
| PFBA |  | 4.17 | 6.25 |  | 102.2 ± 11.1  82.7±8.0 |
| PFPeA |  | 6.67 | 10.00 |  | 98.4 ± 8.7  103.9±12.2 |
| PFHxA |  | 4.17 | 6.25 |  | 98.9 ± 5.5  106.1±20.2 |
| PFHpA |  | 0.83 | 1.25 |  | 109.5 ± 4.9  107.5±6.7 |
| PFOA |  | 4.17 | 6.25 |  | 97.9 ± 10.4  107.7±10.3 |
| PFNA |  | 4.17 | 6.25 |  | 111.1 ± 10.9  110.9±12.3 |
| PFDA |  | 4.17 | 6.25 |  | 111.5 ± 12.1  113.6±8.5 |
| PFUnA |  | 4.17 | 6.25 |  | 101.3 ± 9.1  102.3±16.4 |
| PFDoA |  | 4.17 | 6.25 |  | 99.7 ± 6.0  77.3±12.7 |
| PFTrDA |  | 4.17 | 6.25 |  | 87.1 ± 4.0  ------ |
| PFTeDA |  | 4.17 | 6.25 |  | 78.2 ± 5.5  58.4±10.4 |
| PFBS |  | 0.67 | 1.00 |  | 97.2 ± 1.2  119.7±13.9 |
| PFPeS |  | 6.67 | 10.00 |  | 95.0 ± 8.2  ------ |
| PFHxS |  | 0.67 | 1.00 |  | 117.1 ± 8.5  122.7±30.0 |
| PFHpS |  | 1.67 | 2.50 |  | 112.2 ± 8.7 |
| PFOS |  | 1.67 | 2.50 |  | 96.8 ± 11.6  128.7±7.9 |
| PFNS |  | 1.67 | 2.50 |  | 97.9 ± 23.7  ------ |
| PFDS |  | 0.83 | 1.25 |  | 76.3 ± 7.8  ------ |
| 4:2 FTS |  | 1.67 | 2.50 |  | 83.2 ± 1.7  129.7±2.6 |
| 6:2 FTS |  | 4.17 | 6.25 |  | 112.4 ± 10.6  93.8±21.9 |
| 8:2 FTS |  | 4.17 | 6.25 |  | 91.6 ± 2.5  79.2±15.4 |
| PFOSA |  | 4.17 | 6.25 |  | 92.7 ± 0.2  94.8±19.3 |
| N-MeFOSAA |  | 4.17 | 6.25 |  | *  * |
| N-EtFOSAA |  | 1.67 | 2.50 |  | *  * |

^a^ LCS: laboratory control samples (n=2), prepared by empty vials spiked with 100 μL of 50 μg/L 24 PFAS spiking solution; i.e., 5 ng of each compound.

* Extraction standard recovery < 30%, can’t be quantified.

**Table S6** PFAS concentrations analyzed for the method validation, where the nominal concentration was 16.7 ng/g dw in the goldfish and 25 ng/g dw in the green lettuce.

|  |  | **Goldfish (ng/g dw)** | |  | **Green lettuce (ng/g dw)** | |
| --- | --- | --- | --- | --- | --- | --- |
| **Analyte** |  | ELAP | TU |  | ELAP | TU |
| PFBA |  | 14.0±1.3 | 17.7±2.0 |  | 23.8±1.2 | 24.1±2.4 |
| PFPeA |  | 13.4±0.9 | 17.6±0.5 |  | 20.4±1.7 | 25.3±0.5 |
| PFHxA |  | 16.7±1.1 | 19.3±0.9 |  | 24.5±0.4 | 24.9±1.7 |
| PFHpA |  | 16.8±0.2 | 18.1±1.5 |  | 23.7±1.2 | 25.9±4.0 |
| PFOA |  | 17.4±0.7 | 18.1±1.1 |  | 25.3±1.9 | 24.9±0.5 |
| PFNA |  | 17.5±0.6 | 16.6±2.7 |  | 23.0±3.1 | 24.9±1.4 |
| PFDA |  | 15.9±0.3 | 16.1±0.4 |  | 20.3±3.1 | 21.0±0.4 |
| PFUnA |  | 16.5±0.6 | 18.7±1.4 |  | 22.2±1.9 | 23.8±2.0 |
| PFDoA |  | 16.7±0.5 | 17.6±0.3 |  | 23.3±0.8 | 24.5±4.3 |
| PFTrDA |  | 8.6±0.9 | 15.4±0.9 |  | 16.5±1.2 | 20.5±3.4 |
| PFTeDA |  | 19.0±0.9 | 16.2±2.8 |  | 27.2±1.8 | 24.3±3.0 |
| PFBS |  | 14.9±1.1 | 17.6±2.8 |  | 20.7±0.2 | 22.7±1.6 |
| PFPeS |  | 14.8±0.3 | 18.1±1.4 |  | 19.5±0.5 | 24.2±1.0 |
| PFHxS |  | 11.4±0.5 | 18.6±2.1 |  | 18.9±1.4 | 23.7±5.2 |
| PFHpS |  | 14.0±0.5 | 18.0±1.2 |  | 21.9±0.3 | 26.5±4.7 |
| PFOS |  | 15.9±2.0 | 19.4±1.6 |  | 20.5±1.7 | 22.1±2.3 |
| PFNS |  | 13.9±1.3 | 15.6±3.8 |  | 22.0±3.8 | 22.1±1.8 |
| PFDS |  | 9.0±0.2 | 13.8±2.3 |  | 20.9±1.9 | 18.3±1.7 |
| 4:2 FTS |  | 14.6±1.8 | 13.7±2.5 |  | 20.1±3.3 | 25.8±2.5 |
| 6:2 FTS |  | 15.7±0.9 | 21.9±1.0 |  | 21.7±1.9 | 31.2±0.2 |
| 8:2 FTS |  | 14.8±2.0 | 15.5±1.3 |  | 19.3±1.1 | 28.2±2.9 |
| PFOSA |  | 15.8±0.6 | 16.1±1.1 |  | 22.9±0.5 | 24.4±2.6 |
| N-MeFOSAA |  | 16.9±3.2 | * |  | 27.5±0.7 | * |
| N-EtFOSAA |  | 18.0±0.7 | * |  | 26.8±0.9 | * |

* Extraction standard was not detected or recovery < 30%.

Gray highlights analyzed concentration < 70% or > 130% of nominal concentrations.

**Table S7** Extraction standards recovery (%) analyzed for the method validation.

|  |  | **Goldfish** | |  | **Green lettuce** | |
| --- | --- | --- | --- | --- | --- | --- |
| **Extraction Standard** |  | ELAP | TU |  | ELAP | TU |
| MPFBA |  | 114.3±10.2 | 70.0±4.2 |  | 112.3±4.6 | 88.8±4.5 |
| MPFPeA |  | 81.1±5.1 | 76.0±4.9 |  | 87.1±5.9 | 108.6±8.7 |
| MPFHxA |  | 58.9±1.7 | 53.2±3.8 |  | 62.4±1.1 | 106.4±5.3 |
| MPFHpA |  | 67.9±2.8 | 62.8±4.7 |  | 72.1±6.2 | 109.5±9.5 |
| MPFOA |  | 75.1±0.5 | 64.3±8.5 |  | 69.6±5.0 | 111.7±4.3 |
| MPFNA |  | 73.0±2.0 | 73.7±10.5 |  | 60.4±2.6 | 117.7±9.5 |
| MPFDA |  | 71.9±1.8 | 78.9±3.1 |  | 52.5±3.9 | 121.3±9.0 |
| MPFUnA |  | 59.7±3.4 | 70.4±3.6 |  | 55.1±3.8 | 106.6±4.8 |
| MPFDoA |  | 40.4±2.6 | 60.8±5.1 |  | 53.9±4.0 | 82.1±9.0 |
| MPFTeDA |  | 9.6±1.1 | 42.3±2.9 |  | 22.6±3.2 | 63.0±5.5 |
| MPFBS |  | 75.6±6.3 | 103.7±8.7 |  | 75.6±2.9 | 137.5±7.1 |
| MPFHxS |  | 86.8±1.6 | 100.1±4.3 |  | 83.6±6.4 | 124.8±14.6 |
| M8PFOS |  | 78.4±1.6 | 110.1±18.4 |  | 68.2±4.4 | 137.6±7.2 |
| M_4:2 FTS |  | 68.1±9.3 | 209.6±19.2 |  | 70.7±11.2 | 127.9±7.6 |
| M_6:2 FTS |  | 100.6±4.7 | 207.2±20.3 |  | 70.0±6.2 | 109.0±18.1 |
| M_8:2 FTS |  | 120.7±9.9 | 220.5±29.6 |  | 78.4±0.9 | 106.3±8.0 |
| MPFOSA |  | 43.9±1.9 | 79.7±15.0 |  | 41.5±2.3 | 116.4±11.1 |
| M-NMeFOSAA |  | 64.1±5.7 | 29.6* |  | 60.6±3.4 | 29.5* |
| M-NEtFOSAA |  | 64.1±4.8 | 25.4±5.5 |  | 65.1±2.4 | 24.8* |

* Only detected in one sample, the other two samples were not detected.

Grey highlights extraction standard recovery < 50% or > 150%.
