# Supplementary material for: A modified QuEChERS sample processing method for the determination of per- and polyfluoroalkyl substances (PFAS) in environmental biological matrices
Source: MethodsX. 2023 Jul 20;11:102290. doi: 10.1016/j.mex.2023.102290 (PMC10413343; doi:10.1016/j.mex.2023.102290)
Supplement: Supplementary file 2 [file mmc2.docx]

**Additional Information**

Xiaoyan Yun^a^, Marie J Kurz^b,c^, Rominder Suri^a^, Erica R McKenzie^a, *^

^a^ Civil and Environmental Engineering Department, Temple University, Philadelphia, PA, 19122, USA

^b^ Academy of Natural Sciences of Drexel University, Philadelphia, PA, 19103, USA

^c^ Environmental Sciences Division, Oak Ridge National Laboratory, Oak Ridge, TN, 37831, USA. This manuscript has been authored by UT-Battelle, LLC, under contract DE-AC05-00OR22725 with the US Department of Energy (DOE). The publisher acknowledges the US government license to provide public access to these results in accordance with the DOE Public Access Plan (<https://energy.gov/downloads/doe-public-access-plan>).

^*^**Corresponding author:** Erica R McKenzie, Civil and Environmental Engineering Department, Temple University, Philadelphia, PA, 19122, USA

E-mail address: [ermckenzie@temple.edu](mailto:ermckenzie@temple.edu)

**General background**

Per- and polyfluoroalkyl substances (PFAS) have caused increasing concern due to their potential adverse effects on human and ecological health [1]. To comprehensively evaluate PFAS occurrence and effects in the environment, it is necessary to determine PFAS concentrations in a wide range of matrices, including biota. However, PFAS quantification in biological samples is extremely challenging due to the complexity of the biological matrices, which typically contain proteins, lipids and various co-extractives, which interfere the separation and ionization of the targeted analytes [2, 3].

Alkaline digestion is considered an effective extraction method for biological samples, but the hydrolysis also caused intensive co-eluting interferences [4-6]. Traditional sorbents, such as graphitized carbon and solid phase extraction cartridges, do not achieve effective cleanup to remove interference. The QuEChERS (quick, easy, cheap, effective, rugged, and safe) method was originally developed to determine pesticides from agricultural products, and the method versatility allowed its application in a broader set of contaminants and was employed by various scholastic fields, including environmental topics and biological samples [7]. Previous studies demonstrated that the modified QuEChERS is a promising method for PFAS sample processing, though some inconsistences or contrary conclusions were observed based on published studies [4, 8-12]. Examples of differences in the published literature include the acidification of the organic extraction solvent, the composition and effectiveness of cleanup sorbents.

**Methods comparison**

The published methods for PFAS sample processing in environmental biological samples are mostly based on three extractions: 1) ion pair extraction, 2) solid liquid extraction, 3) alkaline digestion. Extraction procedures are generally followed by cleanup procedures by ENVI-Carb or/and solid phase extraction with Weak anion exchange cartridge (SPE-WAX) [13, 14]. Depending on specific matrix and targeted analytes, different methods present specific advantages and disadvantages. It is difficult to compare the performance of the sample processing methods based on literature, due to the various matrices, different extracted sample mass and analysis specifications (e.g., instrument configuration and sensitivity); reported analyte recoveries are often corrected based on extraction standards and/or injection standards. Therefore, a general comparison and summary of each method is provided herein.

1) Ion pair extraction was developed for PFAS analysis in blood and was used for some aquatic biota and biological tissues [13,15]. However, the method has been shown low efficiency for biological tissues compared to alkaline digestion for some compounds [16]. In addition, this method was used without a clean-up procedure to remove co-extracted interreference, which create an analytical shortcoming [13]. Thus, this method is infrequently used in recent publications.

2) Solid liquid extraction, typically executed with an organic solvent, has been commonly used for PFAS extraction from biota. Acetonitrile facilitates protein and lipid precipitation; therefore, it is commonly used as the liquid solvent for biological samples. However, bulk fats such as triacylglycerols are not soluble in acetonitrile and can result in clogging of the cleanup cartridges [4]. Additionally, it was previously reported that this extraction approach has limited efficiency across the wide range of PFAS (e.g., C4-C14). In our method development efforts (i.e., S1 Previous tested methods), we also observed acetonitrile extracts clogged the SPE cartridges and had low recoveries for short chain PFAS.

3) Alkaline digestion had the best recovery efficiency compared to ion pair extraction and solid liquid extraction. Biological samples were digested with sodium hydroxide or potassium hydroxide methanol solution overnight as the first step [17]. The good performance was attributed to alkaline hydrolysis releasing the conjugated or bonded compounds from the biological samples [4, 16]. However, the vigorous extraction also caused intensive co-extracted interferences and higher matric effects [4, 18]. Compared to other sample processing methods, alkaline digestion is time-consuming [13]. The complex sample processing procedures increase the chance of PFAS loss and contamination of the final extracts [13].

**Conclusions**

Overall, the modified QuEChERS sample processing method is effective for PFAS determination in complex matrices. Although this method has limitations for some PFAS compounds (most precursors: FTSs, PFOSA, N-MeFOSAA and N-EtFOSAA), its excellent performance for commonly detected PFAAs (PFCAs, C4-C14; PFSAs, C4-C10) were evidenced by good extraction standard recovery across a range of varied biological matrices. Compared with other sample processing methods, the modified QuEChERS method is straightforward, easy to transfer, quick, and provides stable performance among various matrices. Due to the wide differences of PFAS compounds and biological matrices, it is challenging to develop a method suitable for all compounds and matrices. However, future development and optimization is promising based on the flexibility of the QuEChERS method to expand analytes and matrices.

**References**

[1] Burkhard, L.P., Evaluation of published bioconcentration factor (BCF) and bioaccumulation factor (BAF) data for per‐ and polyfluoroalkyl substances across aquatic species. Environmental Toxicology and Chemistry, 2021. 40(6): 1530-1543. <https://doi.org/10.1002/etc.5010>.

[2] van Leeuwen, S.P.J., et al., Extraction and clean-up strategies for the analysis of poly- and perfluoroalkyl substances in environmental and human matrices. Journal of Chromatography A, 2007. 1153(1-2): 172-185. <https://doi.org/10.1016/j.chroma.2007.02.069>.

[3] Niu, Z., et al., Recent advances in biological sample preparation methods coupled with chromatography, spectrometry and electrochemistry analysis techniques. TrAC Trends in Analytical Chemistry, 2018. 102: 123-146. <https://doi.org/10.1016/j.trac.2018.02.005>.

[4] Lacina, O., et al., Simple, high throughput ultra-high performance liquid chromatography/tandem mass spectrometry trace analysis of perfluorinated alkylated substances in food of animal origin: milk and fish. Journal of Chromatography A, 2011. 1218(28): 4312-4321. <https://doi.org/10.1016/j.chroma.2011.04.061>.

[5] Valsecchi, S., et al., Determination of perfluorinated compounds in aquatic organisms: a review. Analytical and Bioanalytical Chemistry, 2013. 405: 143-157. <https://doi.org/10.1007/s00216-012-6492-7>.

[6] Al Amin, M., et al., Recent adcances in the analysis of per- and polyfluoroalkyl substances (PFAS) – A review. 2020. 19: 100879. <https://doi.org/10.1016/j.eti.2020.100879>.

[7] Santana-Mayor, Á., et al., Current trends in QuEChERS method. A versatile procedure for food, environmental and biological analysis. TrAC Trends in Analytical Chemistry, 2019. 116: 214-235. <https://doi.org/10.1016/j.trac.2019.04.018>.

[8] Lanza, H.A., et al., Temporal monitoring of perfluorooctane sulfonate accumulation in aquatic biota downstream of historical aqueous film forming foam use areas. Environmental toxicology and chemistry, 2017. 36(8): 2022-2029. <https://doi.org/10.1002/etc.3726>.

[9] Gao, Y., et al., Simultaneous determination of legacy and emerging per-and polyfluoroalkyl substances in fish by QuEChERS coupled with ultrahigh performance liquid chromatography tandem mass spectrometry. Analytical Methods, 2018. 10(47): 5715-5722. <https://doi.org/10.1039/C8AY01478G>.

[10] Zhou, Y., et al., Determination of 20 perfluoroalkyl substances in greenhouse vegetables with a modified one-step pretreatment approach coupled with ultra performance liquid chromatography tandem mass spectrometry (UPLC-MS-MS). Chemosphere, 2019. 227: 470-479. <https://doi.org/10.1016/j.chemosphere.2019.04.034>.

[11] Scordo, C.V.A., et al., Optimization and validation of a method based on QuEChERS extraction and liquid chromatographic–tandem mass spectrometric analysis for the determination of perfluoroalkyl acids in strawberry and olive fruits, as model crops with different matrix characteristics. Journal of Chromatography A, 2020. 1621: 461038. <https://doi.org/10.1016/j.chroma.2020.461038>.

[12] Campbell, K.S., et al., Quantification of PFAS in oyster tissue using a rapid QuEChERS extraction followed by UPLC-MS/MS analysis. Analytical Letters, 2023. <https://doi.org/10.1080/00032719.2023.2208692>.

[13] Valsecchi, S., et al., Determination of perfluorinated compounds in aquatic organisms: a review. Analytical and Bioanalytical Chemisty, 2013. 405: 143-157. <https://doi.org/10.1007/s00216-012-6492-7>.

[14] Carrizo, J.C., et al., PFAS in fish from AFFF-impacted environments: Analytical method development and field application at a Canadian international civilian airport. Science of The Total Environment, 2023. 879: 163103. <https://doi.org/10.1016/j.scitotenv.2023.163103>.

[15] Nakata, H., et al., Perfluorinated contaminants in sediments and aquatic organisms collected from shallow water and tidal flat areas of the Ariake Sea, Japan: Environmental fate of perfluorooctane sulfonate in aquatic ecosystems. Environmental Science & Technology, 2006. 40(16): 4916-4921. <https://doi.org/10.1021/es0603195>.

[16] Taniyasu, S., et al., Analysis of fluorotelomer alcohols, fluorotelomer acids, and short- and long-chain perfluorinated acids in water and biota. Journal of Chromatography A, 2005. 1093(1-2): 89-97. <https://doi.org/10.1016/j.chroma.2005.07.053>.

[17] Shi, Y., et al., Probing the differential tissue distribution and bioaccumulation behavior of per- and polyfluoroalkyl substances o varing chain lengths, isomeric structures and fuctional groups in crucian carp. Environmental Science & Technology, 2018. 52(8): 4592-4600. <https://doi.org/10.1021/acs.est.7b06128>.

[18] Gao, Y., et al., Simultaneous determination of 21 trace perfluoroalkyl substances in fish by isotope dilution ultrahigh performance liquid chromatography tandem mass specctrometry. Journal of Chromatography B, 2018. 1084: 45-52. <https://doi.org/10.1016/j.jchromb.2018.03.008>.
